# Supplementary material for: Do Patients Comply With 12‐h Lithium Blood Level Timing? Findings From a Controlled Clinical Trial and a Real‐World Clinical Setting
Source: Bipolar Disord. 2025 Sep 4;27(7):519–26. doi: 10.1111/bdi.70060 (PMC12620334; doi:10.1111/bdi.70060)
Supplement: Supplementary file 1 — Table S1: Presenting the time between the last supposed lithium intake and the lithium blood test depending on calendar year of the lithium blood test. [file BDI-27-519-s001.docx]

**Supplementary Table 1: Presenting the time between the last supposed lithium intake and the lithium blood test depending on calendar year of the lithium blood test.**

|  | 2012 | 2013 | 2014 | 2015 | 2016 | 2017 | 2018 | 2019 | 2020 | 2021 | 2022* |
| --- | --- | --- | --- | --- | --- | --- | --- | --- | --- | --- | --- |
| Number of samples | 1612 | 2038 | 2997 | 3787 | 5056 | 5673 | 6006 | 6765 | 7144 | 7192 | 3800 |
| Mean time (SD) between last supposed lithium intake and lithium blood test | 14.51 (0.09) | 14.61  (0.08) | 14.62  (0.07) | 14.55  (0.06) | 14.75  (0.05) | 14.53  (0.05) | 14.58  (0.05) | 14.35  (0.04) | 14.26  (0.04) | 14.40  (0.04) | 14.32  (0.06) |
| percentage of lithium blood tests taken <10 or >14 hours after the last supposed lithium intake | 52.11  % | 50.10  % | 48.62  % | 50.49  % | 47.55  % | 47.61  % | 48.82  % | 51.34  % | 52.53  % | 51.35  % | 53.47  % |

Abbreviations: SD = Standard deviation.

* In 2022, data were available until July 31
